# Dextromethorphan Versus Dextrorphan: A Quantitative Comparison of Antitussive Potency Following Separate Administration of Metabolite

**DOI:** 10.1002/jcph.70049

**Published:** 2025-05-16

**Authors:** Saeed Rezaee, Caroline E. Wright, Alyn H. Morice, Amin Rostami‐Hodjegan

**Affiliations:** ^1^ Centre for Applied Pharmacokinetic Research, University of Manchester Manchester UK; ^2^ School of Pharmacy, Zanjan University of Medical Sciences Zanjan Iran; ^3^ Respiratory Medicine, Centre for Clinical Science, Hull York Medical School Hull UK; ^4^ Certara Predictive Technologies Sheffield UK

**Keywords:** antitussive, dextromethorphan, dextrorphan, relative potency

## Abstract

To assess the antitussive effects of dextrorphan (DOR) relative to its parent compound, dextromethorphan (DEX) a double‐blind, randomized, placebo‐controlled crossover study was conducted in 23 healthy volunteers using citric acid cough challenge test after administering placebo, DEX, or DOR. Plasma concentrations and cough frequency were monitored over 24 h, followed by model independent analysis and pharmacokinetic‐pharmacodynamic (PKPD) modelling to discern the relative potency of each moiety. Model‐independent pairwise analysis of the area under the effect curve (AUEC₀₋₂₄ _h_) showed no significant difference between DOR, DEX, and placebo's antitussive effects (*P* >  .06), indicating the influence of considerable inter‐individual variability and the need for larger sample sizes. The model‐based analysis established DOR's relative potency at 26% compared to DEX, with maximum cough inhibition of 23% and IC50 of 0.3 ng/mL. PKPD measures were more accurate for DEX than DOR, particularly at lower baseline cough counts. In conclusion, while DOR retains some antitussive potency, since it is substantially less potent than DEX, higher relative concentrations are required to reach the same effect. Although separate administration of metabolite on its own is considered gold standard to establish its relative potency compared to parent compound, the variability in effect may prevent clear demonstration of effects without modelling particularly when these take benefit of the perturbing the balance of parent/metabolite ratios (e.g. via inhibition) or using the natural variational of such ratios in different individuals.

## Introduction

Dextromethorphan (DEX), a codeine analogue devoid of opiate side effects, is broadly used as an over‐the‐counter cough suppressant.^1^ Although the efficacy of DEX has been confirmed in both clinical^2^, ^3^, ^4^, ^5^, ^6^ and experimental cough challenge studies,^7^, ^8^ there is a paucity of information about the relative contribution of its metabolite dextrorphan (DOR) to overall antitussive activity in humans.

Suggestions that DOR has antitussive activity date from studies conducted in 1953 using unanesthetized dogs.^9^ In an animal study using guinea pigs, it was shown that part of the antitussive activity of DEX may be attributed to its metabolite, DOR.^10^ Additionally, a study conducted on guinea pig brain slices demonstrated that both DEX and DOR possess antiepileptic activity.^11^ Studies in humans have attributed the abuse liability of DEX to its metabolite DOR^12^ and consider this compound to be responsible for neuromodulatory, antinociceptive,^13^ and anticonvulsive^14^ effects. It has been implied that a lack of CYP2D6 activity or a bypass of hepatic first‐pass metabolism using appropriate dosage forms may, therefore, have implications for therapeutic uses.^15^ In another animal study, Fossati et al. showed that DOR exerts a good antitussive activity comparable to that of DEX, but with better tolerability and lower toxicity. Therefore, it is not surprising to see advocacy for the administration of DOR on its own as an antitussive instead of DEX.^16^

Different methods have been used to distinguish between the activity of the parent drug and its metabolite in clinical settings (see Discussions for further details). Our group used a physiologically based PK/PD model to assess the relative antitussive potency of DEX and DOR using the study arms that involved suppressing the inhibition of DEX conversion to its metabolite DOR by quinidine, negating the necessity of DOR administration on its own. DEX is rapidly and extensively transformed to DOR through O‐demethylation by CYP2D6. DOR then undergoes glucuronide formation.^17^ *N*‐demethylation to 3‐methoxymorphinan also occurs, by CYP3A4, but with contributions from CYPs 2C9 and 2C19.^18^, ^19^, ^20^, ^21^ Both DOR and 3‐methoxymorphinan are further metabolized to 3‐hydroxymorphinan by CYPs 3A4 and 2D6, respectively.^22^

Using the previous modeling analysis of interaction study, it was shown that DOR possess 38% relative potency compared to DEX.^23^ However, since many investigators consider separate administration of the metabolite on its own as the gold standard for establishing its potency, the current report assess the relative antitussive potency of DOR using administration of the metabolite on its own for the first time. This was in a separate arm of a study that involved administration of DEX as well as placebo in all individuals who went through citric acid cough challenge test.

## Methods

### Subjects and study design

The clinical trial has been performed according to the Declaration of Helsinki and was approved by the North Sheffield Research and Ethics Committee (approval number NS98/183). All participants enrolled into the clinical trial have given informed consent. Data were collected from twenty‐three healthy non‐smoker volunteers (12 male) aged 19‐51 years who took part in a double‐blind randomized placebo controlled cross‐over study. All participants were genotypically confirmed as normal metabolizers for CYP2D6 and exhibited a forced expiratory volume in one second (FEV1) exceeding 80% of the predicted value. The exclusion criteria were respiratory tract infection or acute cough within the last 4 weeks, receiving any medication within previous 2 weeks, and any medical condition which may interfere with the study. Preceding the visit day, the volunteer was asked to fast for 8 h, abstain from alcohol, and abstain from caffeinated drinks throughout the study day. Written informed consent was obtained from all volunteers. The study was approved by the South Sheffield Research and Ethics Committee. Subjects' cough response to citric acid challenge test was studied on three separate occasions (separated at least 7 days apart) following administration of (i) placebo; (ii) 40.5 mg dextromethorphan hydrobromide; (iii) 44.6 mg of dextrorphan‐D‐tartrate. The order of treatments (placebo, DEX, and DOR) was randomized across participants using a balanced Latin square design to ensure equal distribution of sequence effects. Blood samples for PK analysis were taken periodically (at 0, 1, 2, 3, 4, 5, 6, 8, 10, 12, and 24 h post administration of DEX and DOR).

### Citric Acid Cough Challenge Test

Details of the cough challenge test were described previously.^24^ In summary, cough was produced by five inhalations of 3 mL 10% w/v citric acid (placed in a nebulizing chamber) over 5 min. Prior to randomization, all volunteers underwent a two‐part screening process. Prior to randomization, all volunteers underwent a two‐part screening process. Volunteers who coughed between 7 and 14 times following five inhalations of a 10% citric acid solution at the first visit, were considered eligible for further screening. The second visit evaluated both interday and intraday cough response consistency. The acceptable level of variability in cough frequency was established as no more than 20% between separate days and within a single day across three separate measurements. The frequency of cough response was measured at baseline (prior to placebo or drug administration, t = −1 h) and at regular intervals up to 24 h. Cough measurements were obtained at the same time points as blood sampling.

### DEX and DOR Assay in Plasma

Plasma concentrations of DEX and unconjugated DOR were determined using high‐performance liquid chromatography with fluorometric detection according to the method of Chen et al.^25^ The intra‐assay coefficient of variation for these analyses at a concentration of 2.5 ng/mL ranged from 6% to 13%. The lower limits of quantification were determined to be 0.3 ng/mL for DOR and 0.5 ng/mL for DEX.

### Model‐Independent Analysis of Antitussive Effect

The percent of change in cough number relative to the baseline (t = 0) was plotted against time and the maximum reduction in cough response (E_max_) and its onset time (TE_max_) were determined. The area under the percent of reduction in cough number against time between 0 until 24 h (AUEC_0‐24 h_) was calculated using the trapezoidal rule. The correlation between AUEC_0‐24 h_ and area under plasma concentration time between zero and 24 h (AUC_0‐24 h_) of DEX, (endogenously) formed DOR and directly administered DOR was statistically examined using Spearman rank correlation test. The Friedman test (followed by Dunn's multiple comparison) was used to compare the AUC values of DEX and DOR, as well as the AUEC values, between the different arms of the study.

### Model‐Based Pharmacokinetic/Pharmacodynamic (PKPD) Data Analysis

Pharmacokinetic/pharmacodynamic analysis (PKPD) of DEX and DOR was conducted using non‐linear mixed effects modelling with Monolix software, 2024R1 (Lixoft SAS, Antony, France). The analysis followed a sequential approach, where the PK parameters of both compounds were estimated initially, and then these were used alongside the cough data to build the PK/PD model. The model components are described below:

#### Pharmacokinetic Model

**Figure 1:**
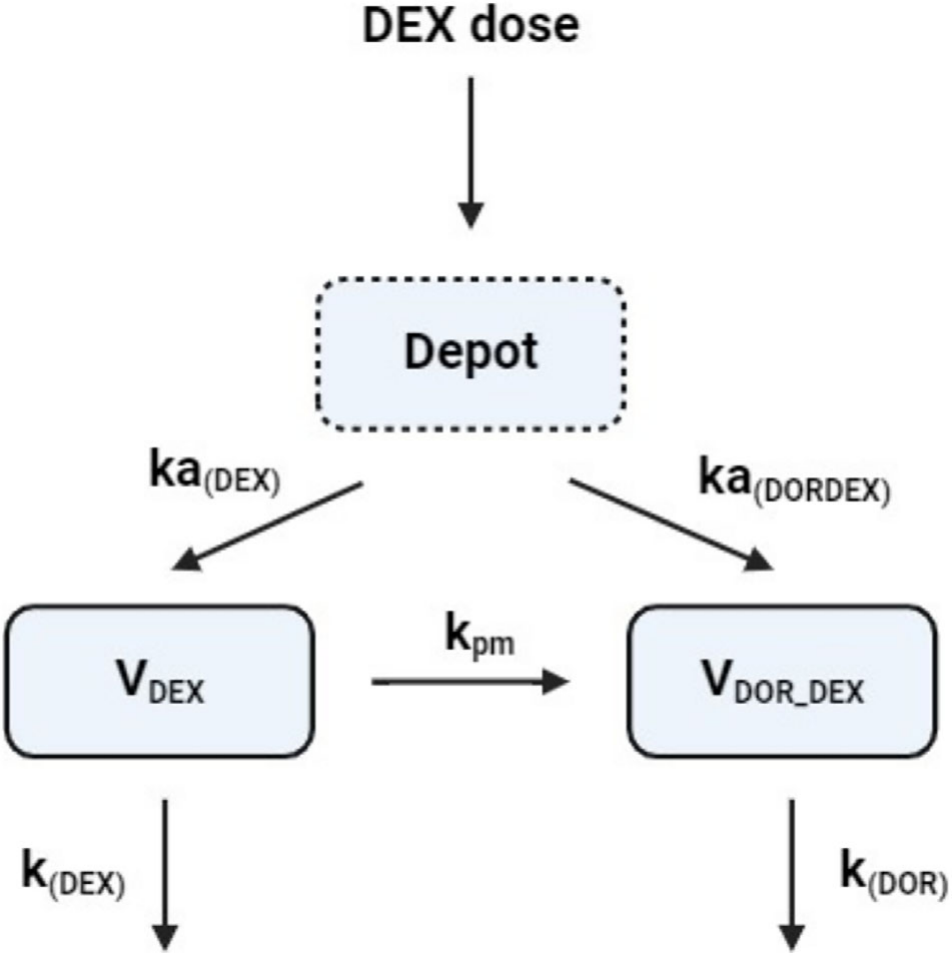
Schematic representation of the joint parent drug‐metabolite structural model with first‐pass effect and without dose‐apportionment used to describe the pharmacokinetics of dextromethorphan and dextrorphan following administration of dextromethorphan. F, bioavailability of the parent drug; DEX dose, dose of dextromethorphan; ka_(DORDEX)_, absorption constant of dextromethorphan; ka_(DOR)_, absorption constant of dextrorphan; k_pm_, transformation rate constant from parent to metabolite; V_DEX_ and V_DOR_DEX_ are the volume of the central compartment for the dextromethorphan and dextrorphan, respectively which are considered to be equal; k_(DEX)_, elimination rate constant for dextromethorphan by other pathways; k_(DOR)_, the elimination constant of dextrorphan.

A joint parent drug‐metabolite model^26^ (Figure 1) was considered for DEX and DOR following administration of DEX with first‐pass effect and without dose apportionment. Different goodness‐of‐fit factors, such as corrected Bayesian information criterion (BICc), various diagnostic plots, such as visual predictive check used for final model selection. This model assumed a one‐compartment model for both compounds and unidirectional transformation of the parent drug to the metabolite in DEX arm of the study. The distribution of DOR after its administration on its own in a separate arm of the study was considered to follow a two‐compartment model. Inter‐individual variability of PK parameters was assumed to be log‐normally distributed. Proportional error model was used to model the intra‐individual variability. For all sub‐models the plasma concentration below the limit of quantitation were left censored in the model.

#### Cough Response Placebo Model

The cough response (number of coughs) of the placebo arm of the study was described by the following equation that has been reported in a previous study^27^ with some minor modifications:

$$E_{\mathrm{Placebo}}=\mathrm{Baselin}e_{\mathrm{cough}}\;\times \;\mathrm{scale}\;\times \;k\;\times \;e^{-k\times t}$$

in which E_Placebo_ is the placebo effect, Baseline_Cough_ is the number of coughs at time (t) zero (before administration of any of the drugs or placebo), scale is the constant of the magnitude of the cough response, and k is the first‐order rate constant for nonlinear suppression of the cough response and return to the baseline. The distribution of all parameters was assumed to be log normal. A constant error model was used to describe intra‐individual variability.

#### PK‐PD Link Model

An effect compartment model in which k_e0 (DEX)_ and k_e0 (DOR)_ are the effect‐site equilibration rate constants for DEX and DOR, respectively, was used to link the plasma concentrations of DEX and DOR to the combined effect of the two moieties at the same receptor. This combined effect is described by the following equation:

$$E\;=E_{\mathrm{Placebo}}\times \left[1-\frac{I_{\max}\times {\left(\frac{Ce_{\mathrm{DEX}}}{\mathrm{IC}50}+\frac{Ce_{\mathrm{DOR}}\times \mathrm{Pot}}{\mathrm{IC}50}\right)}^{n}}{1+{\left(\frac{Ce_{\mathrm{DEX}}}{\mathrm{IC}50}+\frac{Ce_{\mathrm{DOR}}\times \mathrm{Pot}}{\mathrm{IC}50}\right)}^{n}}\right]$$

in which *I*_max_ is the maximum antitussive effect, IC50 the concentration of drugs that results in half of the maximum effect, Pot is the relative potency of DOR to DEX and Ce_DEX_ and Ce_DOR_ are the effect compartment concentrations of DEX and DOR, respectively.^23^ Log‐normal distribution model was used to describe the inter‐individual variability of the model parameters and intra‐individual variability was described by a constant error model.

## Results

### Model‐Independent Analysis of the Antitussive Effect

**Figure 2:**
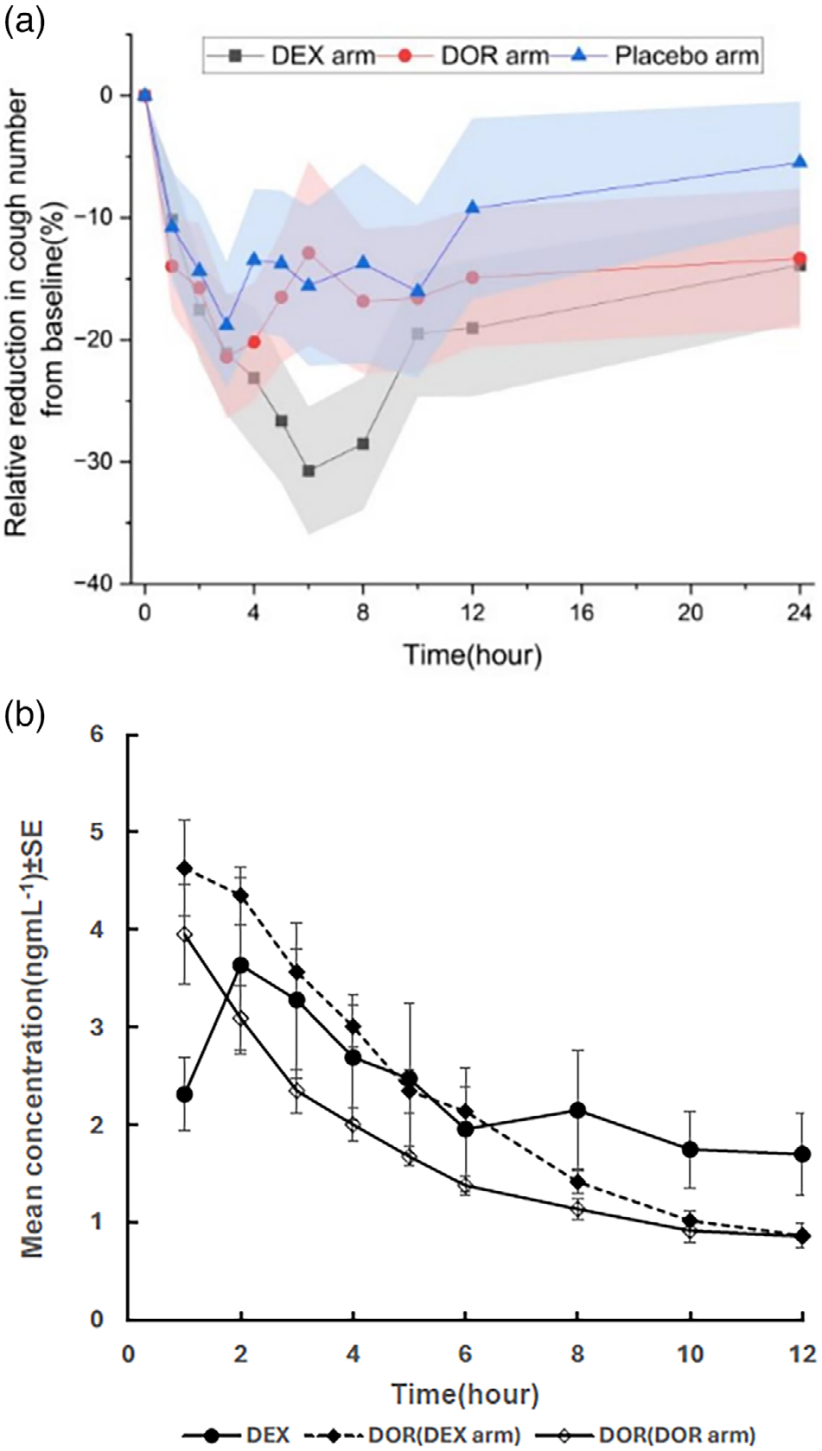
(a) Observed relative change of cough number from baseline in different arms of the study (the shaded areas represent ±SE around the percent of reduction in cough response relative to baseline). (b) Plasma concentration time profile of dextromethorphan (DEX) and dextrorphan (DOR) following administration of DEX and DOR. Error bars represent ± standard error (SE) of mean plasma concentrations.

**Figure 3:**
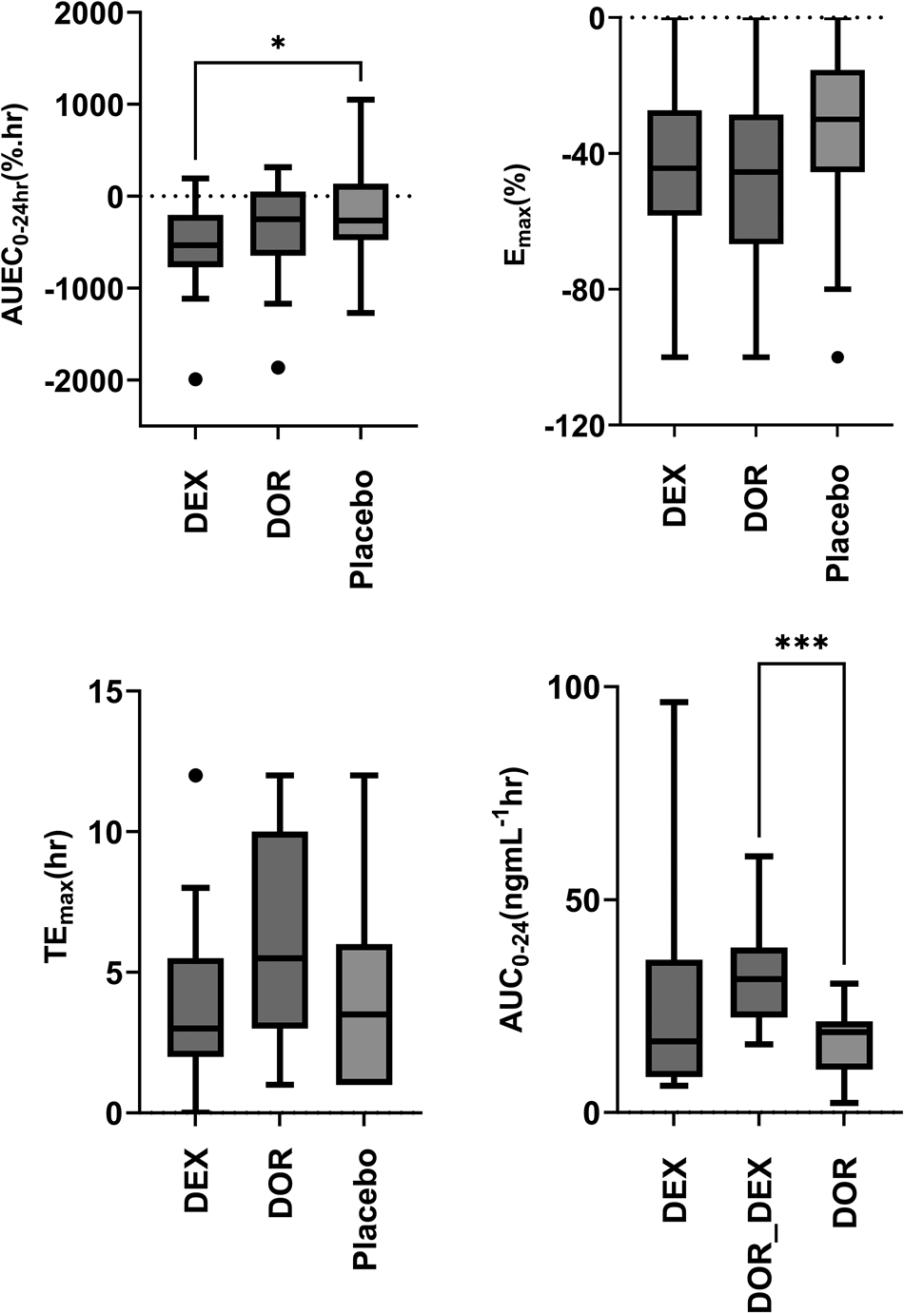
Comparison of parameters used in model‐independent evaluation of dextromethorphan (DEX) and dextrorphan (DOR) anti‐tussive effects (*P‐value =  .055, ***P‐value =  .003)

The relative reduction in cough frequency from baseline across the three study arms is shown in Figure 2a. A statistical summary of the model‐independent pharmacokinetic and pharmacodynamic parameters is provided in Table 1. According to the results of the Friedman and Dunn's tests, the AUC_0−24 h_ of formed‐DOR produced from DEX metabolism was significantly higher than that of direct administration of DOR (*P*‐value =  .003), as shown in Figure 3, despite the fact that a higher molar dose of DOR was administered compared to DEX. Other PK parameters, including C_max_ and T_max_ were not statistically different.

**Table 1:** Descriptive Statistics of Parameters Used in Model‐Independent Analysis of DEX and DOR Antitussive Effect

	Mean	SE	Q1	Median	Q3
**E_max_ (%)**					
DEX	−46	5	−58	−44	−27
DOR	−45	6	−67	−46	−29
Placebo	−32	5	−46	−30	−15
**TE_max_ (h)**					
DEX	3.9	0.7	2.0	3.0	5.5
DOR	6.3	0.9	3.0	5.5	10.0
Placebo	4.4	0.8	1.0	3.5	6.0
**AUEC_0‐24 h_ (%hr)**					
DEX	−299	52	−410	−286	−118
DOR	−189	39	−335	−170	0
Placebo	−138	65	−293	−105	0
**T_max_ (h)**					
DEX	1.8	0.3	1.0	1.0	2.0
DOR (from DEX)	1.5	0.3	1.0	1.0	2.0
DOR	1.1	0.1	1.0	1.0	1.0
**C_max_ (ng/mL)**					
DEX	3.5	0.8	1.3	2.2	4.7
DOR (from DEX)	5.3	0.4	4.1	4.9	6.7
DOR	4.1	0.5	2.7	3.8	6.0
**AUC_0‐24 h_ (ng/mL h)**					
DEX	26.7	6.4	8.3	16.7	35.8
DOR (from DEX)	32.5	2.4	22.4	31.4	38.7
DOR	17.4	1.7	10.1	18.9	21.4

SE, standard error; Q1 and Q3, first and third quartiles; E_max_, maximum percentage of cough number reduction; TE_max_, time of maximum percentage of reduction in cough number; AUEC_0‐24 h_, area under the curve of cough number against time between 0 and 24 h; T_max_, time of maximum plasma concentration; C_max_, maximum plasma concentration; AUC_0‐24 h_, area under the plasma concentration vs time between 0 and 24 h.

Although the Friedman test indicated a significant difference in the integrated cough suppression effect, expressed as AUEC₀₋₂₄ _h_, among the three study arms (*P*‐value <  .05), Dunn's multiple comparison test showed no significant difference between DEX, DOR, and placebo. There were no significant differences in the maximum anticough effect (E_max_) or its time of occurrence (TE_max_) after the administration of DEX, DOR, or placebo(Figure 3).

Spearman test results revealed no significant correlation between exposure and response. Thus, AUEC_0−24 h_, TE_max_ and E_max_ for DEX or DOR with the model‐independent pharmacokinetic parameters of the active moieties.

For the placebo arm of the study, a significant correlation was observed between E_max_ (ρ = −0.6, *P*‐value <  .05) and AUEC_0−24 h_ (ρ = −0.5, *P*‐value <  .05) with age.

### Model‐Based Analysis

**Figure 4:**
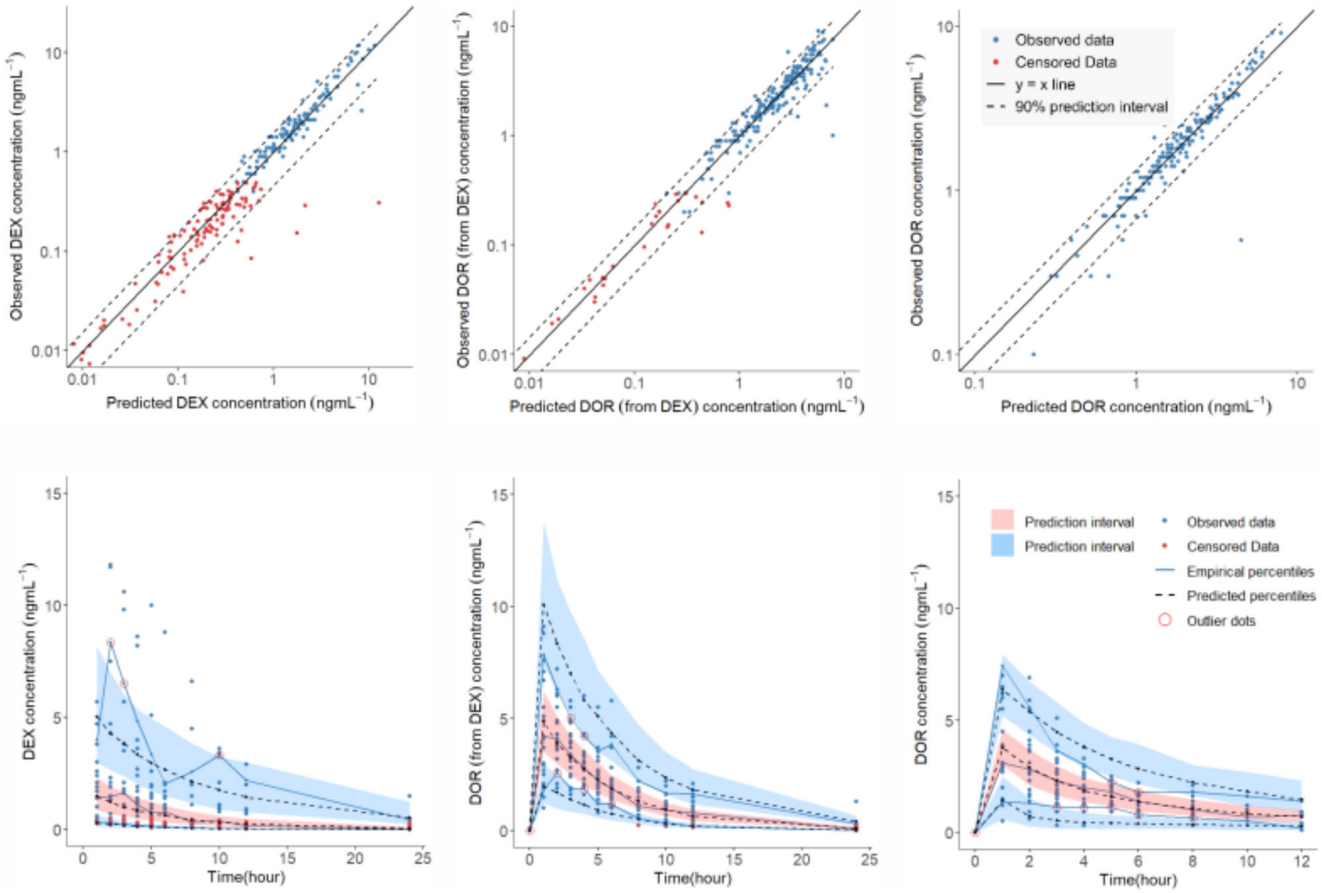
Observed versus predicted (upper panel) and visual predictive check of a combined pharmacokinetic model describing the plasma concentration profiles of dextromethorphan (DEX) and dextrorphan (DOR).

Average plasma concentration‐time profiles of DEX and unconjugated DOR in the two arms of the study are presented in Figure 2b. Since in almost all subjects (except in 2 cases), the 24‐h concentration of DEX and DOR were below the limit of quantitation, the graphs are show up to 12 h. As shown, the concentration of DOR formed by the metabolism of DEX is higher than that of the DOR after preformed DOR is administered on its own. The joint parent drug‐metabolite model with first‐pass effect and without dose apportionment best described the plasma concentration‐time profile of DEX and unconjugated DOR following administration of DEX. The two‐compartment model fitted well to the data of DOR from the administration of preformed DOR. The goodness‐of‐fit of the combined models could be seen in the observed versus predicted plots and visual predictive check graphs of the models (Figure 4). Inclusion of inter‐occasion variability (IOV) on parameters common to both DEX and DOR arms did not improve model performance based on objective function value or parameter precision, and was therefore not retained in the final model. Since there was not enough data in the absorption phase, the value of first‐order absorption rate constant for both DEX and preformed DOR was fixed to the reported values of 2.6 h^−1 23^. To prevent the identifiability problem, equal extravascular apparent volume of distributions were considered for the parent drug and the metabolite in the joint model. It is important to emphasize that the primary focus of this study is on characterizing the PKPD relationship rather than on developing a detailed PK model. While a mechanistic PK model can provide insights, our primary objective can also be achieved using any empirical PK model, provided it adequately describes the data and supports the PKPD link. The selection of a one‐compartment model for DEX and a two‐compartment model for DOR reflects this practical approach and ensures compatibility with the data under different conditions. The disparity of the disposition model for DOR may indicate that formation kinetics from DEX masks the first phase of distribution when assessing the kinetics of formed DOR, whilst this becomes evident under direct administration of DOR. The PK parameters of the combined models are presented in Table 2.

**Table 2:** Population Parameters of the Pharmacokinetic Model Describing Plasma Concentration Profiles Following Administration of DEX and DOR

Parameter	Estimate	%RSE
ka_(DEX)_ (h^−1^)	2.6 (fixed)	‐
ka_(DORDEX)_ (h^−1^)	9.1	28
V/F(L)	4874	10
k_(DEX)_ (h^−1^)	0.1	36
k_(DOR)_ (h^−1^)	0.2	8
k_pm_ (h^−1^)	0.04	44
V_1_/F_(DOR)_ (L)	6984	10
k_a(DOR)_ (h^−1^)	2.6 (fixed)	‐
k_12(DOR)_ (h^−1^)	0.57	39
k_21(DOR)_ (h^−1^)	0.60	44
**Between‐subject variability (CV%)**		
k_a(DORDEX)_	198	18
V/F	48	16
k_(DEX)_	108	31
k_(DOR)_	32	18
k_pm_	43	64
V_1_/F_(DOR)_	32	22
k_12(DOR)_	196	26
k_21(DOR)_	299	26
**Residual error**		
Proportional _(DEX)_	0.3	8
Proportional _(DORDEX)_	0.3	6
Proportional _(DOR)_	0.2	7

%RSE, percent of relative standard error; ka_(DEX)_, absorption constant of dextromethorphan; ka_(DORDEX)_, absorption constant of formed dextrorphan; V/F, volume of distribution (for both dextrorphan and dextromethorphan) after administration of dextromethorphan; V_1_/F, volume of the central compartment of dextrorphan after direct administration; k_a(DOR)_, absorption constant of the directly administered dextrorphan; k_pm_, transformation rate constant from parent(dextromethorphan) to metabolite(dextrorphan); k_12_ and k_21_, transfer rate constants of the dextrorphan after direct administration between central(1) and peripheral(2) compartments and vice versa; CV%, percent of the coefficient of variation

**Figure 5:**
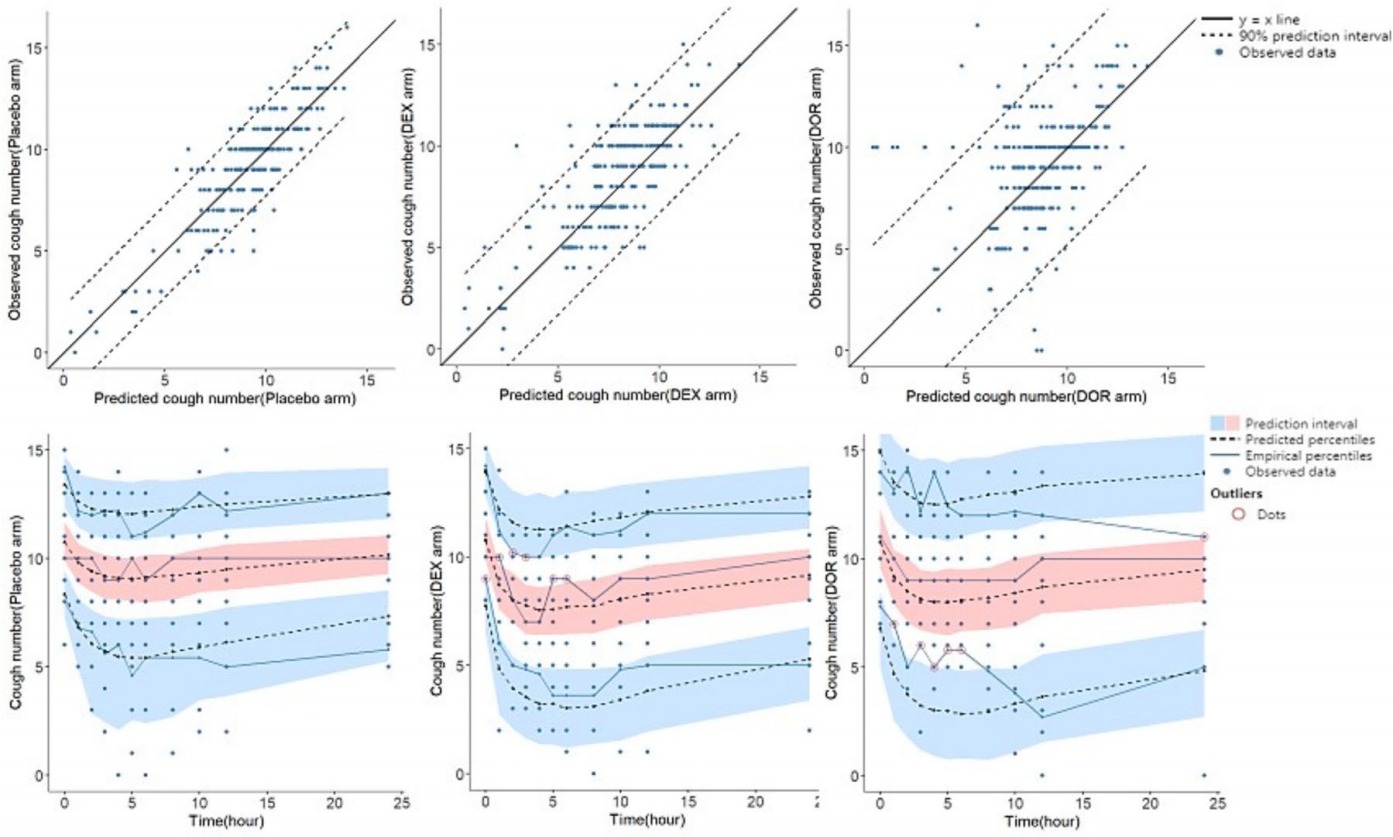
Observed versus predicted (upper panel) and visual predictive check of placebo and PKPD models following dextromethorphan (DEX) and dextrorphan (DOR) administration, describing the cough response‐time profile.

Parameters of the placebo model, which provided an adequate fit to the cough number data collected in the placebo arm of the study, are shown in Table 3. Plots of the observed cough number versus prediction following administration of a placebo, along with the visual predictive check to further verify the validity of the model, are shown in Figure 5. Neither sex nor age was found to significantly influence any model parameters.

**Table 3:** Population Parameters of the Placebo and Pharmacodynamic Models

Parameter	Estimate	%RSE
Baseline_cough	10.7	3
scale	4.5	32
K (h^−1^)	0.2	29
k_e0(DEX)_ (h^−1^)	0.08	110
k_e0(DOR)_ (h^−1^)	0.02	344
IC_50_ (ng/mL)	0.3	327
I_max_	0.23	27
N	2.8	98
Relative potency	0.26	96
**Between‐subject variability (CV%)**		
Baseline_cough	14	18
Scale	115	26
K	85	44
ke_0(DEX)_	176	50
ke_0(DOR)_	171	77
IC_50_	107,425	20
I_max_	68	24
N	205	63
Relative potency	27	902
**Residual error**		
Additive_(Placebo)_	1.4	5
Additive_(DEX)_	2.0	5
Additive_(DOR)_	2.9	5

%RSE, percent of relative standard error; Baseline_cough, the number of coughs before administration of any of the drugs or placebo; scale, constant of the magnitude of the cough response; k, the first‐order rate constant for nonlinear suppression of the cough response and return to the baseline; k_e0(DEX)_ and k_e0(DOR)_, transfer rate constants of dextromethorphan and dextrorphan from the central to the effect compartment; IC_50_, concentration of active moiety in the effect compartment that is associated with half of the maximum antitussive effect; I_max_, the maximum antitussive effect (fraction of the baseline); n, Hill coefficient; CV%, percent of the coefficient of variation.

Parameters of the population pharmacodynamic model are also shown in Table 3. The model estimated the relative potency of DOR to be 0.26 compared to DEX, suggesting that the metabolite retains approximately one‐quarter of the parent drug's potency. Maximum inhibitory effect (I_max_) and IC50 were estimated at 23% and 0.3 ng/mL, respectively. Most of the model parameters showed considerable interindividual variability. The model appears to predict the antitussive effect of DEX (DEX) more accurately than DOR (DOR), particularly for lower to moderate cough counts. For DOR, the model shows more variability and less accuracy, especially at higher observed cough numbers. It seems that the model overestimates the antitussive effect in DOR arm of the study.

Comparing the VPC plots shows that the model generally predicts the observed cough number rather well, as the observed medians mostly fall within the prediction intervals. The comparison suggests that the model fits the DEX data better than the DOR data, particularly at later time points. The DOR plot implies more discrepancies between empirical and theoretical intervals, indicating that the model may be less accurate in predicting the observed data for this treatment arm.

## Discussion

This study provides the first clinical evaluation of the antitussive effect of DOR following its separate administration in humans, enabling a direct comparison with its parent compound, DEX. While model‐independent analysis did not reveal a statistically significant difference in antitussive response among DEX and DOR, or between DOR and placebo—likely due to variability and lower DOR exposure—model‐based PKPD analysis estimated that DOR has approximately 26% of the antitussive potency of DEX. The analysis also identified an IC_50_ of 0.3 ng/mL and a maximum cough inhibition of 23% for DOR. Although DOR is less potent than DEX, its higher systemic concentrations following DEX administration suggest it contributes meaningfully to the overall antitussive effect.

Administration of the metabolite alone and comparison of the effect with that of parent drug were used to compare the hypoglycaemic effect of glibenclamide and its two active metabolites.^28^ Similarly, the auditory and electroencephalographic effects of midazolam were compared to its metabolite, α‐hydroxy‐midazolam, in healthy volunteers.^29^ However, separate administration of metabolite faces many challenges, including but not restricted to synthesizing the metabolite at levels than can be used for clinical studies. Hence many other alternative approaches are also considered to discern the relative activity of the metabolite compared to parent compound.

One approach is to investigate the effect of metabolic polymorphism on the ratio of metabolite to parent drug and the resulting pharmacological effect. Jonkers et al. compared the antagonistic effect of metoprolol and its α‐hydroxy metabolite on terbutaline‐induced hypokalemia between normal and poor metabolizers of CYP2D6.^30^ To assess the relative inhibitory effect of tolterodine and its 5‐hydroxy metabolite (produced by CYP2D6) on salivation, the relationship between the sum of unbound concentrations of the parent drug and the metabolite was compared with the effect observed in poor and normal metabolizers.^31^

PK/PD model‐based analysis of the inter‐ and intra‐individual variability of concentration‐time profiles of both parent drug and metabolite was also used to assess the relative contribution of dihydrocodeine and its metabolite dihydromorphine to analgesia following dihydrocodeine administration by Webb et al.^32^

Selective inhibition of metabolic pathways leading to metabolite formation can be employed as another strategy to elucidate the relative contributions of parent drugs and their metabolites to pharmacological effects.^24^, ^33^

In an attempt to estimate the relative antitussive potency of DOR to DEX, this study considered the separate administration of the metabolite on its own to humans for the first time. The decision on the dose of the DOR was based on the assumption that the metabolism of the “preformed” DOR (this is the molecule that is arriving in liver as DOR) during hepatic extraction will be similar to those of the “formed” DOR (this is the molecule that arrives into liver as DEX but gets converted to DOR during passage through the liver), and a near complete conversion of DEX to DOR. However, comparison of the plasma DOR concentration‐time profiles (and AUC) of metabolically formed DOR from DEX and those of administration of DOR on its own revealed that the exposure to DOR was lower after separate administration, even at a higher molar dose compared to administration of DEX. The term “preformed” DOR, as used in the context of current discussions, is a well‐known concept in drug metabolism that refers explicitly to entry of DOR to liver as DOR (whether following direct administration of DOR on its own, or as DOR in the systemic circulation that was formed in previous cycles of DEX going through the liver). The latter distinguishes such “preformed DOR” from the DOR molecules “formed;” within liver from what entered to liver as DEX. Whilst well‐stirred liver models assume no differences n the fate of “preformed” versus “formed” metabolites, other models which involved zonal distinction of enzymes consider differences depending on the sequence of events that happen to the parent compound and formed metabolites. It has been suggested that the PK of a preformed metabolite might differ from those of a metabolite produced endogenously due to several factors.^34^ Interested readers are referred to reviews on this subject and references therein.^34^ Our observations were an indication of higher hepatic metabolism of “preformed” DOR compared to DOR that is “formed” from DEX during its passage through the liver in the same cycle. It should be noted that some of these differences can be attributed to disparities between parent and metabolite regarding hepatocyte permeability, transporter affinity in addition to zonal/topographical locations of enzymes responsible for secondary metabolism once the primary metabolite is formed. The zonation of the uridine diphosphate glucuronosyltransferase (UGT) enzymes involved in the liver first‐pass metabolism of DOR (as preformed DOR, or as formed metabolite from DEX) is relevant in this respect and has already been reported.^35^

Although specific human data on the oral bioavailability of DOR are lacking, its high water solubility (>10 mg/mL)^36^ and physicochemical similarity to DEX suggest efficient gastrointestinal absorption similar to DEX. This is further supported by data from in vitro studies using Caco‐2 monolayers, which report comparable permeability between DEX and DOR.^37^ Thus, considering the solution form used in this study, formulation‐related differences in absorption are unlikely to account for the lower systemic exposure (after dose normalization) observed following oral administration of DOR on its own, relative to the exposure after metabolic formation from DEX. Hence, we consider differences in hepatic first‐pass metabolism, particularly in light of liver zonation and the differential enzymatic processing of “formed” versus “preformed” DOR as the most likely reason for disparities, knowing that such scenarios are well recognized in the literature.^34^

With the lower exposure to DOR in the arms that involved direct administration of DOR, model‐independent analysis failed to show any statistical difference in antitussive effect between DOR and placebo administration. Also, the antitussive effect from the arm involving administration of DEX was significantly higher than that of the placebo and not different than that of DOR arm. The observed high inter‐individual variability in the PD effect, coupled with the inherently noisy nature of cough responses, highlights the need for larger sample sizes in cough clinical trials to achieve robust results. Consistent with this, attempts to model inter‐occasion variability (IOV) did not improve model performance, further supporting that inter‐individual differences were the predominant source of variability.

Unlike the model‐independent analysis that combines various elements to assess an overall outcome, model‐informed analysis puts emphasis on individual aspects of profiles and obtains additional information from these variabilities. Moghaddamnia et al.^23^ were successful in their model‐based analysis for teasing out the relative DEX and DOR antitussive effects. They reported and IC_50_ value of 3.2 ng/mL the total concentration of DOR (both conjugated with glucuronic acid and unconjugated). This is different than that in the present study of 0.3 ng/mL, which is based on unconjugated DOR. The currently estimated relative potency of 26% is in general agreement with the value derived by their group, 38%, considering that they did not have a separate arm for administration of DOR on its own.

It should be noted that while concentrations were reported in mass units (ng/mL), the molecular weights of DEX and DOR differ by only about 5%, making conversion to molar units unlikely to alter the interpretation of our findings. Such conversion may be more critical when larger discrepancies in molecular weights exist between parent compounds and their metabolites.

Another apparent shortcoming in our analysis relates to modeling the cough counts. Although cough counts are technically discrete outcomes, their high frequency in experimental cough models allows for practical treatment as continuous variables with minimal impact on model validity. This approach aligns with previous studies in the field^38^ and facilitates comparison with earlier works that adopted similar methods.^23^, ^24^ While count‐based models may offer theoretical advantages, our approach prioritized methodological consistency to enable meaningful interpretation within the established literature.

Obviously, the overall antitussive effect from parent and metabolite after administration of DEX is a hybrid outcome from the relative potency of the metabolite (28%‐38% of parent) and the relative concentrations (up to twofold higher for DOR vs DEX, the majority of measurement times following administration). Therefore, lower potency should not be taken as lower contribution of the DOR to the overall effect after administration of DEX.

As a side observation, unlike the previous report on placebo effect being sex‐dependent,^27^ we could not discern any sex effect in the dynamics of the placebo response. Moreover, there was no support for a lag‐time in placebo response in our data. However, the maximum relative reduction in cough number and the integrated relative reduction in cough reduction under placebo administration were directly correlated with age.

## Conclusion

The current study provided data on the antitussive effect of DEX and its active metabolite DOR, which were obtained for the first time by separate administration of DOR to humans, coupled with model‐independent as well as PKPD model‐based data analysis. Results suggest that while DOR might be a significant contributor to the overall antitussive activity of the parent drug DEX, its potency is less than DEX and a higher contribution to overall effects is by the virtue of its higher concentration in the systemic circulation.

The report highlights the complexities of assigning relative potency for parent drug and metabolite and suggests that alternative approaches to administration of the metabolite on its own could be as effective, if not more, in discerning the potency by using perturbation of the relative concentrations of parent and metabolite.

## Conflicts of Interest

The authors declare no conflicts of interest.

## Funding Information

This study was partly supported by a grant from the Procter and Gamble Company.

## Data Availability

Data can be made available on request.
